# Public Abattoirs a Necessity to Public Health

**Published:** 1885-07

**Authors:** 


### Art. XIX.—PUBLIC ABATTOIRS A NECESSITY TO PUBLIC HEALTH.

There is no way of evading the fact that the consumer of articles of food, especially when of an animal nature, should be guaranteed by some competent and trustworthy authority, that the same are not only free from disease, but also free from disease-producing causes.

Every question which has to do with the public weal is of a necessity a political one, and any medical, or other journal, that interests itself in questions of this nature cannot help making them such.

The time will certainly come, when, nobody can tell, when every city will have its abattoir for the slaughter of all and every animal destined for human consumption, even though it be for use in the owners family, and when all such animals will be subjected to veterinary inspection both before and after slaughter. Every town should also have its slaughter-house, and there should be one to each district in the country parts of the State. Not an animal except poultry should be allowed to be slaughtered outside of such slaughter-houses. That every English and American city is far behind many on the continent in this regard, can be easily seen by visiting the Abattoirs at Paris, Berlin, Munich and other cities. These countries have not got the agricultural districts measured off yet. It will be readily seen that such measures are only applicable to thickly settled parts, and not where the farms are large and villages few and far between. Such persons must assume their own responsibility.

We have considered it more conformable to our purpose to offer to our readers the following very freely translated notes from Supt. Dr. Hertwig's report of the workings of the Berlin Abattoir, for the first year of its existence, than to make up a paper from our personal observations. We wish herewith to express our thanks, not only to the Superintendent, but to all his deputies for their universal politeness and kindness.

With the 1st of April, 1883, came into action the law making it obligatory that all animals destined for slaughter in and around Berlin, should be killed at one locality. It is evident that such a law rendered very great changes in the manner of doing business on the part of the butchers, yet it must be said to their credit that they have nearly all seen its benefits to themselves as well as the public. The number of animals slaughtered was: 93,387 cattle, 78,220 calves, 171,077 sheep, 244,343 swine. The number of butchers located at the works during the year was about 567. During the Winter months numerous private persons came to the works to slaughter their own swine. According to the nature of their calling, the butchers may be divided into three classes, viz.: wholesale, retail and contract butchers, or such as do work for others instead of buying animals on their own account.

All the flesh that is here produced is not destined for consumption in and around Berlin alone, much being sent into the interior of Germany, especially pork. From November to June there is a large export of mutton directly to Paris, special cars being built for the purpose, the track leading directly into the house. During the last year, 18,591 sheep were thus shipped to Paris. They are all stamped with a French stamp as if slaughtered in France, the people of Paris having still a strong antipathy to German things.

The days' work at the works is very well indicated by the following selections: One day's record, cattle, 743; calves, 484: sheep, 978; swine, 1,471.

Sundays, Mondays, Tuesdays and Thursdays are the busiest days, but little being done on Saturdays.

The inspection consists of a chief veterinary inspector and ten veterinary assistants; a book-keeper, four stampers or branders, a microscopist, and a veterinarian as supervisor of the meat inspection bureau; four sectional veterinary inspectors, eighty-seven sub-inspectors and thirty persons engaged in collecting specimens from the hogs as they are slaughtered. Each cattle slaughter-house has six veterinary inspectors who have also to do with the respective stables. There are four in the hog slaughter-house, and in the Winter months it is ncessary to increase the number. Ten extra inspectors have been arranged for on three days of the week. All animals are subjected to a most rigid examination both before and after slaughtering.

The diseases which have during the past year led to the condemnation of the entire animal, were: Tuberculosis 182 times, Hog Cholera 72 times,Icterus 38 times, Dropsy 18 times, Unhealthy Appearing Flesh 9 times, Badly Bled 3 times, Putrifaction beginning 1 time, Echinococci 1 time, Measles 1,621 times, Trichiniasis 216 times, Lime deposits in flesh of hogs 19 times, Actinomycosis in pork 15 times.

Single parts or organs were condemned in 21,229 cattle, 86 calves, 4,806 sheep, 7,401 swine. Tuberculosis was found in cattle 2,613 times, in calves 2 times, in swine 1,313 times, which led to the condemnation of 102 carcasses and 4,226 single organs in cattle, 2 calves, 78 swine and 1,940 organs of the latter.

The majority of these animals were more or less emaciated. Whenever the disease had acquired any considerable degree of generalization, the entire carcass is to be condemned. In the case of the calves there was a wonderfully generalized development of miliary tuberculosis of recent origin. In the cattle the "perlsucht" or serosa variety prevailed, while in swine pulmonary tuberculosis was most frequently seen. In cattle, tubercles were found three times in the flesh and single bones, while in swine none were found in the flesh but 56 times in the bones.

Where there has been no generalization of the disease, and where little or no emaciation exists, the flesh is allowed to be sold. (An interesting question is what becomes of the old, diseased, tuberculous cows in America? The poor eat them; drink their milk, and thereby help to hasten off their children. Not one comes to condemnation. I know of several butchers that make a practice of buying these and other diseased animals, and I know this meat is sold in the streets of Boston. The same may be true of every other American city.)

To continue the translation, microscopic examination of the tuberculous material demonstrated the characteristic bacilli to be present. They were also found in the milk from an udder in which there were tuberculor processes.

Dropsy and emaciation caused the condemnation of six cattle, five calves, five sheep and two hogs. In two of these cattle there was found most extensive disorganization of the lungs and liver, and once in cattle and hogs there was a large echinococcus invasion in the lungs; in two cattle and five sheep the livers were badly disorganized by distoma, and in one hog hydrops renalis was present to a great degree. To the diagnosis of Icterus it is necessary that the examination be made by daylight. In some regions the food leads to a yellow tinge to the flesh and fat of animals which cannot be mistaken for Icterus, when we take into consideration the condition of the skin, mucous membranes and other organs. In the case of five swine, the carcasses of which were condemned on account of the unpleasant appearance of the meat, although no disease was present, it is interesting to know that the animals had been fed on fish and oil-cake. The flesh had a peculiar fatty appearance, somewhat resembling smoked salmon, and had a very penetrating fishy smell.

In the revision of the cooling out rooms, there were condemned 81 cattle lungs, 167 sheep lungs, 20 livers from cattle and 64 from sheep, with 53 hearts from the latter, on account of beginning putrefaction. From other causes there were condemned 393 cattle lungs, 3 from calves, 78 from sheep and 805 from swine, and the livers from 291 cattle, 4 calves, 66 sheep and 50 hogs. Thread-worms—strong glids—were found in the lungs of 1,833 swine and 67 sheep.

On account of accumulation of blood in the tissues, 760 kilos of flesh were condemned during the year: 10,504 lungs and livers were condemned on account of echinococci invasion. The increase in size and weight of some of these organs was remarkable; for instance, while the normal liver of an ox weighs from 5 to 6 kilos and is about 35 centimeters long and 25 wide in one of these diseased organs the weight was 28 kilos, the length 75 centimeters, and width 50.

It is frequently stated that the distoma begin to leave the bodies of their hosts in the early Summer months and that the animals are free from these parasites during the real hot months, but examinations have shown that they are present at all times of the year. Abscesses were found in 1,279 lungs and 411 livers; 14,603 cows were found to be with calf in various stages of development; 1,621 swine were condemned on account of measles.

Most of these came from Poland. In some parts of the country it is the custom of the dealers to examine the swine for measles when they are destined for the Berlin market, hence it is that we do not often find them in such hogs. When only one cysticera is found in a swine, then the whole body is divided into the necessary sections, and the meat cut up for sausage purposes, but not hashed, under the supervision of an officer; if a second cysticera is not found then the flesh is not condemned. The carcasses of such animals are treated here on the grounds under police control, the lard is tried out and allowed to be sold, but the flesh and bones are made into phosphates for agricultural purposes. Aside from in the flesh we find the cysticera very frequently in the heart and brain, once in the eye, but not in the liver, lungs and kidneys; 216 hogs were condemned for trichinæ. They seem to be pretty equally divided among all breeds.

The diaphragm, its pillars; the tongue, abdominal muscles, intercostals and those of the larynx of 150 swine were subjected to the per cental observation of their numerical invasion with trichinæ. Ten pieces of each of these muscles, having a diameter of one quarter centimeter, making 9,000 specimens, were thus examined:

| | | | Trichinæ. |
|---|---|---|---|
| In 1,220 | specimens from the muscles of | Diaphragm | 987 |
| " 1,400 | " " | Pillars " | 1,329 |
| " 1,370 | " " | Tongue. " | 1,115 |
| " 870 | " " | Abdominal muscle. | 491 |
| " 730 | " " | Intercostals. | 308 |
| " 1,220 | " " | Laryngial muscles: | 710 |

Hence the pillars of the diaphragm are the most profusely invaded parts of the body, the intercostales the least. The latter fact shows their unfitness for trustworthy examinations. We thus thought to do away with this part of the examination, but the recent discovery of actinomycosis fungi in pork by Inspector Dunker, and the fact that these very muscles are the most profusely invaded by this new and dangerous parasite, renders it necessary that we still give these muscles our most exact attention.

The above-mentioned discovery was made in February, 1884, and during this month and March they were found in the flesh of fifteen swine. The result of these examinations is as follows: A peculiar irritated condition of the tissues—flesh—is to be observed from the presence of the fungi during all its phases of life. They frequently calcify, and their presence can only be made out by appropriate treatment with a diluted acid. Invaded spots of muscles show a shrinking or contraction of the primitive bundles, and granular degeneration of their substance. These changes gradually augment until such spots possess a dirty bronwnish color, and finally the irregular formed fungi becomes developed. These centres consist of a mycelium like staoma, consisting of very dense collections of fine threads which run in every direction, but as they approach the peripheries of the growth assume a more direct course and end in the usual pear-shaped protuberance characteristic of their fungi. The finer details of these fungi are not to be made out save with much circumspection. As soon as they have arrived at complete development they at once begin to calcify, whereby one may see them in a variety of forms which finally become enclosed in a sort of capsule composed of connective tissue.

The real place of these fungi in the natural kingdom has not yet been definitely determined, some authors classifying them with the rust fungi of grain and others with the mould fungi. Those holding the latter view look upon them as the conidia of such fungi which have, through some form of adaptation, acquired the ability to develop in animal tissues. Nothing is known of their manner of existence outside the animal organism, but the fact of their appearance in the mouth and adjacent parts makes it evident that they find the means of their introduction most probably in vegetable food. It is a very important question, how far the duties of a hygienic police have to do with these parasites in the food we eat, especially as we know that fungi of the same appearance occur in connection with a disease having the same name and nature in man. All hog meat, or other, is unquestionably condemned for this cause as an article of food.

The inspection of meat is done most thoroughly, there being a special building set apart for the microscopic examination of all meat, especially pork. There are 87 examiners in this building, and 30 persons are engaged in taking specimens from certain designated parts of the carcass of each animal. In the long months of Winter this number is insufficient, and extra examiners and specimen collectors have to be appointed. They are not appointed until after a most exacting examination, the most difficult and questionable objects being placed before them, and they must also serve a period of approbation before they can be definitely placed as examiners. A large number of these practising as microscopic examiners are women; the same proving a pleasant and paying task for them, they also make very competent inspectors.

Those employed to gather specimens from the slaughtered hogs take 24 specimens from each hog, viz., 6 from the muscles of the diaphragm, 6 from its pillars, 6 from the muscles of the larynx, 6 from the abdominal muscles, each set of 6 specimens are collected in a small tin box with the name of the section of the body stamped in the cover. Large specimens from these are then cut off and placed in order between two heavy glass plates some eight inches long and half inch thick, and are then pressed out very thin by screws which draw the plates together; they can then all be examined at once. These glass plates are divided into sections and numbered; numbers 1 to 6 for the specimens of the diaphragm, etc. Specimens which appear at all doubtful are at once brought to the attention of the chief inspector.

Special instruction upon meat inspection is given to the examiners by Dr. Dunker when it seems necessary.

